# Aging, working memory capacity and the proactive control of recollection: An event-related potential study

**DOI:** 10.1371/journal.pone.0180367

**Published:** 2017-07-20

**Authors:** Jessica Keating, Caitlin Affleck-Brodie, Ronny Wiegand, Alexa M. Morcom

**Affiliations:** 1 School of Psychology, Philosophy and Language Sciences, University of Edinburgh, Edinburgh, United Kingdom; 2 Centre for Cognitive Ageing and Cognitive Epidemiology, University of Edinburgh, Edinburgh, United Kingdom; University of Cambridge, UNITED KINGDOM

## Abstract

The present study investigated the role of working memory capacity (WMC) in the control of recollection in young and older adults. We used electroencephalographic event-related potentials (ERPs) to examine the effects of age and of individual differences in WMC on the ability to prioritize recollection according to current goals. Targets in a recognition exclusion task were words encoded using two alternative decisions. The left parietal ERP old/new effect was used as an electrophysiological index of recollection, and the selectivity of recollection measured in terms of the difference in its magnitude according to whether recognized items were targets or non-targets. Young adults with higher WMC showed greater recollection selectivity than those with lower WMC, while older adults showed nonselective recollection which did not vary with WMC. The data suggest that aging impairs the ability to engage cognitive control effectively to prioritize what will be recollected.

## Introduction

A common complaint of healthy older adults is difficulty remembering events. Although memory seems vivid, they are less likely to recall details, associations, and contextual information [1,2]. A leading theory of cognitive aging attempts to explain these episodic memory impairments in terms of wider difficulties with cognitive control, which result from loss of integrity of prefrontal cortex [3,4]. Age-related differences on memory tasks are particularly marked when a high degree of voluntary control is required [5]. For example, age effects are greater in free recall tasks than when specific cues are given, and are frequently absent for simple item recognition [6]. In part, this pattern can be explained by failures of recollection with preservation of a more automatic but nonspecific sense of familiarity [7,8]. However it is less clear why recollection fails. Control is involved at a number of different stages, and what happens prior to retrieval may be critical [9].

We used electroencephalographic event-related potentials (ERPs) to examine the degree to which older and younger adults prioritized recovery of relevant information from memory according to their current task goals. The ERP left parietal (LP) old/new effect is a positivity elicited by correctly identified studied compared to unstudied items in a recognition test, occurring from around 500–800 ms post-stimulus. An established neural correlate of successful retrieval, it is thought to reflect recollection as opposed to familiarity in recognition memory tasks [10–12]. The magnitude of the left parietal old/new effect for targeted compared to non-targeted information can be used to index the degree of relevant as opposed to irrelevant recollection [13]. Dywan et al. [13] used this approach to investigate the impact of goal-directed control of retrieval in young and older adults in a recognition exclusion task [14]. At test, previously studied items were designated as targets, and repeated items presented only in the test phase, as well as novel items, were designated as non-targets. While the young showed larger parietal old/new effects to targets than familiar non-targets, older adults showed effects of similar magnitude for the targets and non-targets. Further evidence that this reflected failure of attentional control on the part of the older adults was supported by a second experiment which showed that when young adults performed a secondary task at retrieval, their ERPs resembled those of the older adults. These findings suggest that aging affects control operating at or before the point of recollection.

### Aging, proactive cognitive control, and episodic retrieval

Failures of pre-retrieval control in aging may reflect a wider shift from *proactive* to *reactive* cognitive control underpinning impairments in self-initiation of behavior [9]. According to Braver’s ‘dual mechanisms of control’ theory, the proactive mode depends on active maintenance of goal representations in prefrontal cortex which allows behavior to reflect current goals. Proactive control is essential for self-initiated behavior, minimizing interference from competing goals triggered by the environment. Reactive control in contrast is stimulus-driven and transient, and helps to resolve interference once it is present [15,16]. Studies of attentional control suggest that older adults are impaired at proactive control, and rely more on reactive control [17–21]. Proactive control is also closely related to the ability to maintain goal information online in working memory in the presence of interference, as reflected in measures of working memory capacity (WMC; [22–24], see also [25]. Behavioral studies suggest that these proactive control functions may be important in pre-retrieval control of episodic memory: young adults with high WMC are more effective than those with lower WMC at strategically supporting recollection by self-generation of retrieval cues and use of encoding context [26,27]. However, behavioral data do not establish whether these strategies are implemented before, as opposed to after, the point of retrieval. Direct evidence comes from two recent ERP studies. Using the same exclusion task as Dywan et al. [13], Elward and Wilding [28] showed that WMC is associated with selective recollection, finding larger LP old/new effects for targets than for non-targets in individuals with higher WMC. Parietal effect magnitude for non-targets did not vary with WMC, but for targets it was greater in high-control individuals, suggesting that those with lower WMC were less able to focus their recollection on the targeted information. The same was found in a subsequent study using a slightly different exclusion paradigm, in which two different deep encoding tasks—either judging how difficult a word’s referent would be to draw (Artist task) or how many functions they could think of for it (Function task)–served as two alternative sources at test [29]. Items from each task were designated as targets in separate blocks, with items from the other source being designated as non-targets. Critically, one group of participants was given a taxing Stroop task to complete prior to the exclusion task, and this group showed a smaller difference in amplitudes between target and non-target LP effects. This extended the individual differences evidence, causally implicating WMC in the ability to control recollection proactively [30].

There have been no direct investigations of the relation between age effects on proactive control and episodic memory, although some fMRI findings are consistent with a shift to reactive control of retrieval (see [9]). Velanova et al. [31] found delayed BOLD responses in prefrontal cortex in older adults relative to the young. In another study, hippocampal activity in response to preparatory retrieval cues was reduced in older adults while prefrontal activity during retrieval itself was increased, although it was not possible to determine whether this shift was memory-specific [32]. ERP studies of course offer greater temporal precision than fMRI, and Dywan et al.’s findings ([13]; described above) support the notion of an age-related reduction in control at or prior to the point of retrieval, reflected in the selectivity of recollection. It is not yet possible to attribute this to impairment in wider proactive control functions. However, there is converging ERP evidence linking developmental changes in cognitive control to pre-retrieval processing at the other end of the lifespan. A reduced difference between target and non-target left parietal old/new effects has also been shown in adolescents relative to young adults [33], analogous to Dywan et al.’s finding in older adults. Furthermore, individual differences in WMC correlated with this ERP difference measure only in the young adults, who may also have differed from adolescents in this regard. These data suggest that groups in whom proactive attentional control is less effective may fail to engage these resources effectively in the control of recollection.

### The present study

The present study examined how the relation between WMC and recollection selectivity—as indexed by the difference in the magnitude of ERP parietal old/new effect for targets compared to non-targets—differs as a function of age. To avoid confounding age with the overall level of task difficulty, which has been implicated as a factor influencing the selectivity of retrieval [34,35], we matched the level of target hits between age groups. To discourage use of recall-to-reject strategies, our exclusion task also included a pre-study phase in which some items were pre-studied in one task then studied in the other. Therefore, recollection that an item had been studied in the currently non-targeted task was insufficient to make the correct decision that it was not a target (see [36] for a similar manipulation). We expected that, in line with other studies, older adults would show reduced WMC and less selective recollection than the young, replicating Dywan et al.’s finding [13]. We also expected that in young adults, recollection selectivity would be greater in individuals with greater WMC, replicating Elward, Wilding and others’ findings [28,30]. Critically, we predicted that the relation between WMC and recollection selectivity would not be observed in the older adults, converging with findings from other populations with reduced attentional control [30,33], and supporting the proposal that older adults fail to engage attentional resources in order to control recollection. This would also provide further support for the involvement of proactive attentional control in pre-retrieval control of episodic memory.

## Materials and methods

### Participants

Participants were twenty healthy younger (13 female; range = 21–30 years) and twenty-one older (13 female; range = 62–79 years) adults. Young participants were mainly recruited from the University of Edinburgh student community and older participants from the Psychology Volunteer Panel of adults from across the lifespan (http://www.ed.ac.uk/ppls/psychology/research/volunteering). Data from a further nine participants were excluded due to insufficient trials following removal of EEG artefact (2 younger, 3 older) or near-chance performance on the exclusion task (1 younger, 3 older; defined as discrimination between Target and Non-target trials < .20). For the analyses of WMC, 20 young and 16 older adults had valid scores on the O-Span test. Participants took part in two experimental sessions for remuneration. All were right-handed fluent English speakers, had normal or corrected-to-normal vision, and did not report any neurological disorders. Summary data including results of standardized cognitive tests (described in the next section) can be found in Table 1. The study was approved by the Psychology Research Ethics Committee (ref.: 133-1415/8) and data were collected between May and August 2015.

**Table 1:** Summary participant data and standardised cognitive test results. Data are from 20 young and 19 older participants except for O-Span, for which scores were available for 16 (Absolute) and 17 (Partial) older adults (see text for details).

	Younger adults		Older adults	
	M	SD	M	SD
Age	24.0	1.9	68.8	6.0
Years of education	6.93**	1.17	4.71**	3.52
Digit-symbol coding	90.50**	12.82	72.79**	19.81
Trail-making contrast score	10.32	1.43	10.47	2.04
COWAT letter fluency	44.19*	12.15	55.16*	15.45
TOPF VIQ estimate	104.1***	3.51	118.2***	7.05
O-Span				
Absolute	46.95	16.45	37.81	20.94
Partial	61.48	9.00	57.35	12.79
HADS Anxiety	7.77*	3.77	5.00*	2.89
HADS Depression	2.05	1.70	2.79	1.51

Years of education were calculated as full-time equivalent after age 16 years. Significant differences between age groups are indicated by asterisks (* p < .05; ** p < .01; *** p < .001). M = mean; SD = standard deviation.

### Standardized cognitive tests

To assess WMC and its relationship to ERP measures we used a computerized version of the O-Span task [37]. In this task participants complete mental arithmetic operations (e.g., (1x1) + 2 = ?) while remembering letters presented individually in serial order. Trials are in groups of 3 to 7, each contributing one letter. The partial score reflects the number of letters recalled in the correct order, and the absolute score the number of letters recalled from only trial groups that were 100% correct [38]. To characterize the two age groups we also administered several baseline tests (data were not obtained for two older participants). The Test of Premorbid Functioning (TOPF) was used to assess crystallized (verbal) intelligence [39], expected to be spared or improved with age. Executive function and processing speed were measured using the Controlled Word Association Test (COWAT) [40], the Digit-Symbol Coding subtest of the Wechsler Adult Intelligence Scale IV [41,42] and the D-KEFS Trail-Making Test [43]. In addition, we screened participants for mood disorder using the Hospital Anxiety and Depression Scale (HADS; [44]. All but five (4 younger, 1 older) participants scored in the normal range, and none scored greater than two standard deviations below the mean. These tests were administered in a separate experimental session lasting around 45 minutes.

### Recognition memory task

#### Materials

Stimuli for the experimental task were 649 four- to nine-letter nouns (median = 6) were sampled from the MRC Psycholinguistic Database [45]. Mean Kucera-Francis [46] written frequency was 13 per million (range = 1–50), concreteness was 588 (range 550–670) and imageability 577 (range = 490–659). 368 words were randomly selected from this sample for use in the experiment. For each participant, these words were randomly allocated to a pre-study list, a set of study lists, a set of test lists, and start-list fillers. For all participants, the pre-study lists consisted of 48 words (24 to be studied in the Artist and 24 in the Function task). To equate performance across age groups, study and test lists were split into eight cycles for older participants (30-words at study and 42 at test) and six cycles for younger participants (40-words at study and 56 at test) on the basis of pilot testing. Each test block began with two unstudied filler words for which ERP data were not analyzed. Thus each participant responded to 96 target words, 96 non-target words, and 96 new words, as well as 48 pre-studied targets.

#### Procedure

The memory task consisted of pre-study, study and test phases. EEG recording took place during the study and test phases. Study and test phases were separated by intervals of 1–5 min during which participants completed 5 pen-and-paper true or false questions. All stimulus words were presented in the center of a computer monitor in black upper case ‘Arial’ letters against a gray background. Key press responses were registered using a computer keyboard. Participants first completed practice with self-paced and actual-paced versions of the tasks.

The pre-study phase was included in order to discourage use of recall-to-reject strategies by including some pre-studied words studied in both tasks (see Introduction). Pre-studied items were not included in the ERP analysis. The pre-study phase consisted of two mini-blocks in which participants made an Artist decision (‘How easy is it to draw the object?’), indicating their judgements as ‘very easy’, ‘easy’, ‘difficult’ and ‘very difficult’ using button-presses or a Function decision (‘How many functions can you think of for the object’) indicating their judgements as ‘none’, ‘a few’, ‘some’, and ‘a lot’. They were told that these words might be re-presented in the main experiment. All pre-studied words were in fact re-presented in the study phase during the other decision task, and then served as targets in the test phase. At test, participants were explicitly instructed that target items could have been studied during either the pre-study list or the preceding study list.

In the main study phase, participants again made an Artist decision during half the mini-blocks and a Function decision during the other half. Study trials were self-paced with an average maximum stimulus onset asynchrony (SOA) of 7000 ms. Trials started with stimulus presentation (500 ms), followed by a blank screen (100 ms), then a black fixation cross (5000 ms or until participant response, plus a randomly selected interval of 900 ms, 1000 ms, or 1100 ms), and finally a red fixation cross to indicate the next trial was imminent (300 ms), followed by a blank screen (100 ms) before the next trial. The order of Artist and Function mini-blocks was counterbalanced within participants across cycles.

In the test phase, items from one encoding task were designated as targets in each study test cycle (Target-Artist or Target-Function). Participants were instructed to respond ‘yes’ if they had seen the word previously in the target encoding task and ‘no’ to all other words. Items were designated as targets whether they had been studied in the pre-study phase or the study phase. Test trials began with stimulus presentation (500 ms), followed by a blank screen (100 ms), then a black fixation cross (3500 ms regardless of participant response), then a red fixation cross to indicate the next trial was imminent (300 ms), and a final blank screen (100 ms) before the next trial began. The Artist task was targeted in odd-numbered cycles and the Function task in even-numbered cycles. At both study and test, responses were counterbalanced such that half of the participants used A and Z to indicate easier to draw / more functions and M and K to indicate difficult to draw / fewer functions, and vice versa for the other half of the participants.

### EEG recording and preprocessing

Following the practice session and pre-study list, prior to the experiment proper, participants were fitted with an elastic cap housing 64 active silver/silver chloride electrodes in the extended International 10–20 system configuration [47], labelled using Modified Combinatorial Nomenclature [48]. Two additional electrodes—CMS (Common Mode Sense) and DRL (Driven Right Leg)—took on the function of a ground electrode while concurrently supporting electrical noise rejection. Bipolar electrode pairs were placed on the outer canthi and above and below the right eye to record horizontal and vertical EOG, respectively. Electrodes were also placed on the left and right mastoid processes. Using a BioSemi Active Two (http://www.biosemi.com/products.htm) AD-box with 24-bit resolution signal digitization, EEG and EOG were recorded continuously at a 1024-Hz sampling rate with amplifier bandwidth of 0–208 Hz (3 dB). EEG was recorded in relation to the CMS reference electrode, and then re-referenced offline to the average of the mastoid electrodes. A 0.1–30 Hz digitized Butterworth filter was applied (as well as 50 Hz notch filter for channels with line noise present) and then data were down-sampled to 128 Hz and epoched into 2100-ms segments, including a 100-ms pre-stimulus baseline. Segments with excessive muscle artefact, drift, or gross eye movements were rejected following manual inspection. Ocular artefact correction was achieved by removing vertical EOG and horizontal EOG components from the EEG data in an independent component analysis (ICA; [49,50]). For one older and two younger participants, a satisfactory ICA decomposition could not be obtained so blink artefacts were corrected using a linear regression method [51]. Excessively noisy channels were replaced using nearest-neighbor averaging, and individual participant grand-average data were visually checked for residual artefacts.

## Results

Behavioral and ERP data were analyzed for all participants with sufficient artefact-free trials for ERP analysis (N = 20 young and 21 older; see Participants). The principal experimental conditions were correct responses to the three exclusion task trial types: Target Hits (studied items designated as targets attracting correct “target” responses), Non-target correct rejections (CR; studied items designated as non-targets attracting correct “non-target” responses), and New CR (unstudied items attracting correct “non-target” responses). Performance for the pre-studied Target items was analyzed separately, and these items were excluded from the ERP analyses. In all analyses of variance (ANOVAs), degrees of freedom and p-values are corrected for nonsphericity [52]. Where residuals were non-normal, outliers were identified and removed [53].

### Standardized cognitive tests

The results of the standardized cognitive tests are summarized in Table 1. Two measures were computed for WMC, the absolute score and the partial score (see Materials and methods, standardized cognitive tests). Absolute O-Span scores were available for 19 young and 16 older participants, and Partial scores for 20 young and 17 older. Although neither measure differed according to age group on average (*t*(33) = 1.49, p = .146; *t*(35) = 1.16, p = .255), both absolute and partial scores showed clear age effects within the older group (for correlations with age, r = -.54, p = .03; r = -.65, p = .005). The two scores were very strongly correlated both across all participants (r = .90, p < .001) and in the older group alone (r = .88, p < .001). To maximize N for the ERP analyses involving WMC, we used the Partial O-Span scores. In normative studies partial-credit scoring for WM span tasks is generally preferred due to its greater reliability and distributional normality (see [38]). The results of the other statistical comparisons of the other standardized test scores between groups are given in Table 1. Older adults had fewer years of education but higher verbal IQ as estimated using the TOPF. The young adults were also slightly more anxious than the older group as measured using the HADS. The main ERP results were therefore checked using this score as a covariate of no interest. The pattern of findings was unchanged, so this analysis is not reported further. A check analysis using verbal IQ as a covariate is also reported below for the ERP left parietal effect.

### Memory task performance

#### Study phase response times

Accuracy on the Artist and Function tasks could not be assessed since these judgements were purely subjective. We computed response times (RTs) for each study task decision to determine whether the age groups differed in terms of time taken to make these judgements. ANOVA on median RTs with factors of Task (artist/ function) and Group (young/ older) showed that Function decisions took longer across both age groups (for Artist task, M = 1574 ms, SD = 420; for Function task, M = 1782, SD = 420; for main effect of Task, *F*(1,38) = 31.62, MSE = 653560, p < .001, η^2^_p_ = .45; for Task x Group, *F*(1,38) = 2.68, MSE = 55390, p = .110). Older adults responded slightly (but not significantly) more slowly (for main effect of Group, *F*(1,38) = 3.11, MSE = 1099599, p = .086). To check whether time to encode items varied with individual differences in WMC, we also conducted a multiple regression analysis on RTs collapsed over task. Since this was done to check for possible differences in encoding with implications for interpretation of ERP old/new effects (see Discussion: ERP findings) it included the same participants, i.e., excluding two older adults due to outlier values (> 3 SD from the mean) on the O-Span and the ERP measure, respectively. Predictors were Group, O-Span and a Group x O-Span interaction term formed by mean-correcting O-Span scores within groups and multiplying the older group’s scores by -1. Age was also included as a regressor of no interest (see ERP results: Left parietal old/new effect for more detail). This regression model was not significant (*F*(4,40) = 1.72, MSE = 301662, p = .171).

#### Recognition memory accuracy

Table 2 summarizes memory task performance for participants included in the EEG analysis. Trials categorized as possible anticipations due to short RTs were excluded from analysis (< 600 ms; this cutoff was amended to 300 ms for 3 faster-responding young participants; no more than 10 trials were excluded in any participant).

**Table 2:** Memory task performance. Mean accuracy proportions and RTs are shown for each group separately by targeted task and trial type. Mean target-non-target discrimination (D) for each task is also given (SDs in brackets). Note that pre-studied items were always targets (see Materials and methods).

	Target Artist				Target Function			
	Target	Non-target	New	Pre-studied	Target	Non-target	New	Pre-studied
**YOUNG**								
Accuracy	.84 (.10)	.84 (.12)	.98 (.03)	.81 (.15)	.80 (.13)	.87 (.09)	.98 (.04)	.71 (.15)
D		.68 (.18)				.66 (.17)		
RT (ms)	1319 (298)	1481 (348)	1023 (233)	1399 (356)	1471 (287)	1441 (255)	1022 (196)	1534 (352)
**OLDER**								
Accuracy	.89 (.08)	.79 (.13)	.98 (.03)	.88 (.11)	.74 (.14)	.88 (.13)	.98 (.03)	.73 (.20)
D		.68 (.18)				.62 (.17)		
RT (ms)	1620 (267)	2039 (380)	1117 (218)	1756 (454)	1986 (419)	1780 (295)	1094 (171)	1968 (420)

ANOVA on accuracy proportions with factors of currently targeted Task (artist/function), Condition (target hits/ non-target CR/ new CR) and Group (young/older) revealed a 3-way interaction (F(1.3,50.2) = 7.80, MSE = .063, p = .004, η^2^_p_ = .16) as well as a main effect of Condition (*F*(1.7,69.1) = 52.7, MSE = .74, p < .001, η^2^_p_ = .57) and interaction of Task x Condition (F(1.3,50.2) = 25.6, MSE = .21, p < .001, η^2^_p_ = .39). *Post hoc* analysis showed a significant interaction of Task with Condition only in the older group (F(1.2,24.6) = 25.3, MSE = .28, p < .001, η^2^_p_ = .55; for young, F(1.4,27.0) = 3.61, MSE = .018, p = .055), and a significant group difference in accuracy only in the Artist task (for Group x Condition, *F*(1.8,72.6) = 4.45, MSE = .03, p = .018, η^2^_p_ = .10). In the Artist task, the young were equally likely to respond correctly to Targets and Non-targets, but in the older group, Target hits were more frequent than Non-target correct rejections. In the Function task, the groups did not differ reliably (*F*(1.4, 55.2) = 1.34, MSE = .02, p = .26) and Non-target performance was more accurate than Target performance overall (M = .85 and .82). Despite these subtle differences between tasks, however, overall accuracy (across both tasks) did not differ between young and older adults by condition (for Group x Condition *F*(1.7,69.1) = .23, MSE = .00, p = .76).

The Target-Non-target discrimination index D (proportion of target hits—non-target false alarms) did not differ significantly according to age group across tasks (*F*(1,40) = .62, MSE = .01, p = .44; M = .68 in young, .65 in older), but across both age groups D was higher for the Artist task (*F*(1,40) = 4.41, *MSE* = .03, p = .042; M = .68 for Artist, .64 for Function; for interaction, *F* < 1). A planned comparison also confirmed that the groups did not differ in accuracy for novel items according to the currently targeted task (see Table 2; *F* < 1).

#### Recognition memory response times

We examined median RTs for accurate responses to targets, non-targets and novel items in the test phase. ANOVA with factors of Task (artist/function), Condition (target hits/ non-target CR/ new CR) and Group (young/older) revealed a 3-way interaction (F(1.3,50.0) = 11.8, MSE = 414304, p < .001, η^2^_p_ = .23) as well as interactions of Task with Condition (F(1.3, 50.0) = 41.4, MSE = 1456680, p < .001, η^2^_p_ = .52) and Condition with Group (F(1.7,65.0) = 21.5, MSE = 1058902, p < .001, η^2^_p_ = .36) and main effects of all factors. While RTs were generally slower for older adults and for target hits and non-target CR than for new CR, the speed of responses to targets and non-targets depended on task and age group. *Post hoc* tests showed that in the Artist task, target hits were faster than non-target CR in both groups and this difference was greater in the older adults (for Group x Condition, *F*(1.9, 75.3 = 20.3, MSE = 602399, p < .001, η^2^_p_ = .34). In the Function task, target hits were *slower* than non-target CR in the older group only (for Group x Condition, *F*(1.5,58.6) = 14.1, MSE = 712330, p < .001, η^2^_p_ = .27; see Table 2). As for accuracy, planned comparison for New CR alone did not reveal any group differences (for effects of Task, F < 1, for Group main effect, *F*(1,40) = 1.82, MSE = 143587, p = .19).

#### Pre-studied items

As a check on strategy in the exclusion task, we also evaluated memory performance for the pre-studied items (see Table 2), although these were not included in the ERP analysis. Pre-studied items were always targets, but had been studied in both tasks (once in the initial pre-study phase in the currently non-targeted task, then again in the study phase in the currently targeted task; see Recognition memory task procedure). Hit proportions for pre-studied items were corrected by subtracting false alarms to new items (one older participant did not receive any pre-studied target items due to a programming error, and was therefore excluded from this analysis). ANOVA with factors of Group (young/ older) and (artist/ function) Task revealed high accuracy, which was significantly greater in both groups when the Artist task was targeted (for main effect of task, *F*(1,40) = 19.0, MSE = .21, p < .001, η^2^_p_ = .32). There were no differences between age groups (for interaction of Group with Task, *F*(1,40) = 2.47, MSE = .03, p = .124; for main effect, *F* < 1). A further ANOVA with the additional factor of Target Type (Target/ Pre-studied Target) also confirmed that young and older adults did not differ in target accuracy according to whether items had been pre-studied.

Responses were generally slower to pre-studied targets in the Function than the Artist task, consistent with the accuracy data. This effect of task on RT was more pronounced in the older group (*t*(20) = 4.38, p < .001; in young, *t*(19) = 1.86, p = .078). Comparing Targets with Pre-studied Targets, responses to the latter were faster in the older group only (for Group x Target Type, *F*(1,39) = 7.10, MSE = 607038, p = .001, η^2^_p_ = .15; for Target Type in older group, *F*(1,20) = 19.91, MSE = 805893, p < .001, η^2^_p_ = .50).

#### Accuracy by study-test cycle

Because the targeted task alternated every other study-test cycle, it was possible that participants learned to prioritize encoding of the task which would next be targeted, potentially impacting on the available retrieval strategies. However, a breakdown of target-non-target discrimination performance showed no evidence of improved exclusion task performance over the first 3 pairs of cycles in each task (note that young adults only completed 3 pairs). ANOVA with factors of Cycle Pair (1/ 2/ 3), Targeted Task (artist/ function) and Group (young/ older) showed no hint of any effects of Cycle Pair (all *F* < 1; for 1^st^ cycle, young M = .70, SD = .18; old M = .67, SD = .20; for 3^rd^ cycle, young M = .69, SD = .24; older M = .69, SD = .27).

In debrief, participants in both age groups reported that they had followed the incorrect exclusion task instruction in the test phase of one or more cycles (e.g. they targeted Artist words instead of Function words in a target-Function cycle). While it was possible that participants simply had difficulty remembering which task was targeted, reversal of instructions would undermine the exclusion analysis. Prior to EEG data analysis, accuracy data were checked for each cycle for negative values for target-non-target discrimination (D) in all participants. We used a strong criterion for evidence of reversal: a negative D value for a cycle (< -.20) suggesting poorer than chance performance, and no evidence of across-the-board poor performance (the D for the affected cycle should be > .50 below the mean across other cycles). In such cases the codes were swapped for target and non-target items. Where D for a cycle was more than .50 below the mean but greater than -.20 we assumed that there was no consistent targeting of either task, and discounted the cycle from behavioral and ERP analysis. Under these criteria, one cycle was excluded in 1 older participant, one cycle was swapped in 5 older and 4 younger, two were swapped in 1 older, and three were swapped in 1 older. The numbers of participants with one, or more than one, excluded block did not differ reliably between age groups (χ^2^ (1) = .24 and 2.1, p = .24 and .14).

#### WMC and exclusion task performance

Before assessing the relations between ERP old/new effects and working memory capacity, we asked whether WMC predicted performance on the experimental task in the young and older groups. This analysis included all participants with O-Span scores, except for one older with an outlier value (see Study phase response times for details). Using D (target-non-target discrimination) as the dependent measure, we evaluated a regression model with predictors of Group, O-Span, Group x O-Span and Age. The model as a whole did not predict significant variance in D (*F*(4,30) = 1.86, MSE = .06, p = .143). However, when the two age groups were analyzed separately, O-Span reliably predicted discrimination in the young (R^2^ = .59, *F*(1,19) = 9.67, MSE = .16, p = .006; β = .59, *t*(18) = 3.11, p = .006; Bonferroni-adjusted alpha = .025), but not in the older adults (*F*(1,13) = .01, MSE = .00, p = .929). These *post hoc* tests therefore confirmed presence of a significant relation in the young group, but did not clearly establish whether or not there was a between-group difference.

### ERP results

**Fig 1:**
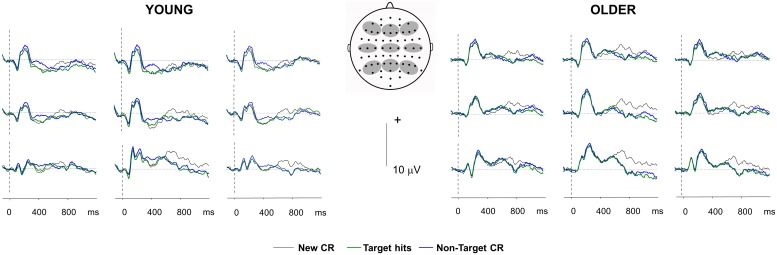
Grand-average ERP waveforms for old/new effects. Waveforms show cross-participant grand-average waveforms pooled over frontal (left: (F3, F5, F7, AF7), midline: (F1, F2, Fz), right: (F4, F6, F8, AF8)), central (left: (C3, C5, T7), midline: (C1, Cz, C2), right: (C2, C4, T8)) and parietal (left: (P3, P5, P7, PO7), midline: (P1, Pz, P2, POz), right: (P4, P6, P8, PO8)) electrode clusters. The 3 conditions included are Target Hits (items studied in the targeted task attracting correct ‘target’ responses), Non-target CR (items studied in the non-targeted task attracting correct ‘non-target’ responses), and New CR (novel items attracting correct ‘non-target’ responses). Positive-going ERPs are plotted upwards. See ERP results section for details of analysis.

Fig 1 shows grand average waveforms for ERPs elicited by Target Hits, Non-target CR, and New CR in young and older adults. The mean numbers of trials (range in brackets) contributing to individual subjects’ ERPs in these three conditions for the young were 60 (34–92), 59 (30–81), and 70 (46–90), respectively, and for the older group, 62 (27–81), 62 (26–86) and 75 (38–94). The mean amplitudes of old/new effects were computed as the average ERP for each subject elicited by Target Hits and Non-target CR, after subtraction of the average ERP elicited by New CR. From around 300 ms until around 600 ms post-stimulus, ERPs to Target Hits and Non-target CR were generally more positive-going than those to New CR. From 300–500 ms these old/new effects had a mid-frontal maximum in the young group and a right frontal maximum in the older group, and in the young were more prominent for Non-target CR than for Target Hits. Between 500–600 ms old/new effects had a left parietal maximum in both groups consistent with presence of a left-parietal (LP) old/new effect. From around 700 ms there was a sustained late posterior negative old/new effect (LPN) in both groups, with negative-going ERPs for Target Hits and Non-target CR relative to New CR. This was maximal at midline parietal sites and did not differ between conditions, continuing until at least 1100 ms. At frontal sites, there was also evidence of a positive-going old/new effect in both age groups, predominantly for targets, with a right frontal maximum, onsetting around 800 ms and continuing until at least 1100 ms post-stimulus. Our main *a priori* hypotheses concerned the LP effect, but in order to clarify the findings, we also examined the early frontal effects, the LPN which overlapped the LP effect, and the late frontal effect, which has previously been associated with control processes acting after the point of recollection [12,54].

#### Left parietal old/new effect

**Fig 2:**
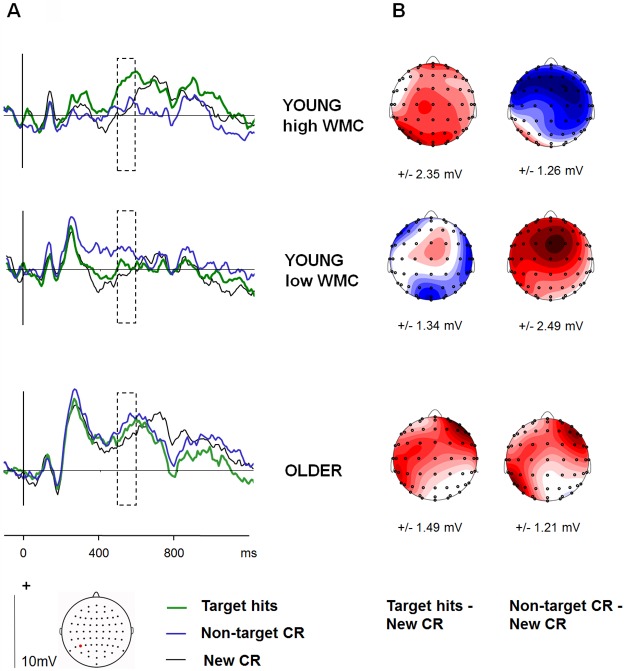
Left parietal old/new effects. (A) Grand-average waveforms at the P5 electrode site for each age group, showing ERPs to the 3 main conditions. The young group are divided into high-WMC and low-WMC sub-groups, based on a median split (see ERP results). (B) Scalp topographies of between-condition differences in mean ERP amplitude from 500–600 ms. The topographic plots are of old-new subtractions for Target Hits and Non-target CR. Maps are scaled to the maxima (red) of each effect, and their ranges are displayed under each map in microvolts.

ERP waveforms and scalp topographies for the left parietal old/new effect are shown in Fig 2, with young participants divided into high-WMC and low-WMC subgroups to illustrate the individual differences within this age group. Following the strategy of Elward et al. [28,30], we examined the magnitudes of ERPs elicited at the left- and right-sided parietal electrode sites P5 and P6 within the typical time window of 500–800 ms [11,12]. Because of the temporal overlap between the expected positive-going old/new effect and the negative-going LPN effect at parietal electrodes, we first checked only Target old/new effects collapsed over age groups to establish when a positive-going LP effect was present at the left (P5) electrode site. The old/new effect was robustly positive from 500–600 ms (for main effect of old/new, *t*(40) = 2.72, p = .010, d = .42) non-significant from 600–700 ms (*t*(40) = -.93) and robustly negative from 700–800 ms (*t*(40) = -3.15, p = .003, d = .49). Therefore, to assess modulations of the positive-going LP effect separately from modulations of the negative-going LPN, further hypothesis-driven analyses at P5 were restricted to this first time window. ANOVA on target old/new effects at P5 and P6 electrode sites with factors of Group (young/ older) and Hemisphere (left/right) revealed a strong left-lateralization across groups (for main effect of Hemisphere, *F*(1,39) = 24.13, p < .001, MSE = 44.5, η^2^_p_ = .38). Effects involving Group were non-significant (*F* < 1). This confirmed presence of an LP effect for targets which did not differ according to age group.

We checked that the scalp distribution of old/new effects during this time-window was comparable in the two age groups and conditions, analyzing range-rescaled data to remove confounding effects of differences in overall amplitude [55], at 15 electrode groupings over the frontal (left: [F3, F5, F7, AF7], midline: [F1, F2, Fz], right: [F4, F6, F8, AF8]), frontocentral (left: [FC3, FC5, FT7], midline: [FC1, FC2, FCz], right: [FC4, FC6, FT8]), central (left: [C3, C5, T7], midline: [C1, Cz, C2], right: [C2, C4, T8]) centroparietal (left: [CP3, CP5, TP7], midline: [CP1, CPz, CP2], right: [CP2, CP4, TP8]) and parietal (left: [P3, P5, P7, PO7], midline: 1, Pz, P2, POz], right: [P4, P6, P8, PO8]) scalp. ANOVA with factors of Group (young/ older), Condition (target/ non-target), anterior-posterior electrode Chain (frontal/ frontocentral/ central/ centroparietal/ parietal) and Hemisphere (left/ midline/ right) did not reveal any Group or Condition differences in the topography of this old/new effect (for interactions of Chain or Hemisphere with these factors maximum *F* = 2.04, all p > .1).

Next, we examined the effects of age and working memory capacity on the LP old/new effect at P5 for Targets and Non-targets. ANOVA with factors of Group (young/ older) and Condition (target/ non-target) did not reveal any average differences between age groups (one older outlier removed; *F* < = 1 for main effects of Condition, and Group and their interaction) but the old/new effect was robust overall (for main effect, *F*(1,39) = 10.24, MSE = 3.9, p = .003, η^2^_p_ = .21).

**Fig 3:**
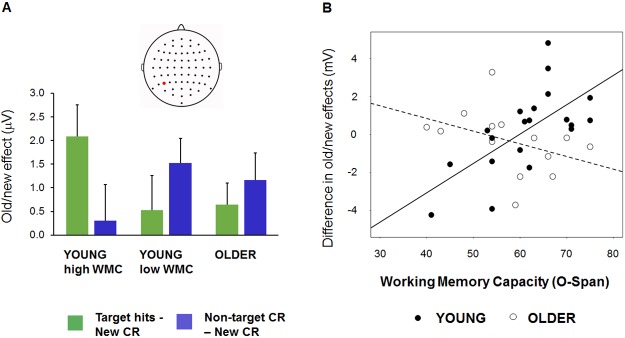
Recollection selectivity and working memory capacity (WMC). Both plots illustrate ERP old/new effects for Target Hits and Non-target CR (i.e., ERPs elicited by each condition after subtraction of ERPs elicited by New CR) at the P5 electrode site in the 500–600 ms time window. (A). Mean amplitudes (and standard errors) of Target and Non-target old/new effects according to age group, in microvolts. The young group are divided into high-WMC and low-WMC sub-groups, based on a median split (see ERP results). (B) Associations between WMC (*x*-axis) and the ERP index of recollection selectivity: the difference in magnitude between target and non-target old/new effects (Target old/new effect—Non-target old/new effect in microvolts; *y*-axis). Individual data points correspond to participants, and lines show the linear regression fits within age groups. See Standardized cognitive tests for details of O-Span measures and ERP results for details of ERP measures.

To test the main test predictions about relations between working memory capacity and the control of recollection, regression analyses were conducted on an ERP difference measure obtained by subtracting LP old/new effects for Non-targets from those for Targets, as an index of the selectivity of the LP effect to targeted material [28]. Two older adults were excluded from analysis due to outlier values (> 3 SD from the mean) for the O-Span and the ERP measure, respectively. Regression models had predictors of Group, O-Span and the interaction of Group x O-Span. The interaction term was formed by mean-correcting O-Span scores within groups and multiplying the older group’s scores by -1. Age was also included as a regressor of no interest. Where age ranges in cross-sectional between-group studies are wide, as here, including age in the model helps to ensure that observed individual differences—in this case, associations between WMC and ERP old/new effects—are not secondary to age variation within groups. This is important because individual differences within older age groups may in fact be due to differences in age, i.e., mean effects of age, rather than individual differences in age effects [56]; see [57]. This was particularly the case in the present study where age was significantly associated with WMC within the older group (see above), indicating a wide range of age and/or ability within this group. Therefore, we wanted to ensure that apparent associations between individual WMC and the LP effect did not simply reflect age effects on the LP effect. The results of the regression analysis are illustrated in Fig 3. The model explained significant variance in target-selectivity of the LP effect (R^2^ = .34, *F*(4,30) = 3.87, MSE = 11.3, p = .012) with a significant interaction of Group with O-Span (β = .54, *t*(33) = 3.126, p = .003). The effect of the continuous variable Age was not significant. As a further check that the interaction of Group with O-Span was not an artefact of ‘regressing out’ age effects within the older group which correlated with WMC, we also ran the model without age as a predictor, and the interaction of Group with O-Span remained highly significant (β = .47, *t*(33) = 3.14, p = .004; for model, R^2^ = .32, *F*(3,31) = 4.84, MSE = 14.2, p = .007). Given the difference between groups in verbal IQ as measured with the TOPF, we also repeated the original analysis with the verbal IQ estimate as an additional predictor of no interest. The results with regard to Group with O-Span were unchanged (for interaction, β = .54, *t*(33) = 3.19, p = .003; for model, R^2^ = .35, *F*(5,29) = 3.09, MSE = 9.2, p = .024). *Post hoc* simple regression analyses on Target and Non-target old/new effects had the predictor O-Span. These showed larger LP effects for Targets in the young adults with greater WMC (R^2^ = .21, *t*(19) = 2.17, p = .04; β = .45), with no such association in the older adults (*t*(14) = .41). Findings for Non-targets were not significant, suggesting that the overall group differences were driven mainly by Target effects. However, separate full models for Targets and Non-targets were non-significant (*F*(4,29) = 1.45, MSE = 6.0, p = .243; *F* < 1). A further *post hoc* analysis in the young group used a median split by O-Span (N = 9 in high-WMC group with Part O-Span > 62; N = 11 in low-WMC group with Part O-Span < = 62). This showed that the positive target-non-target difference in the LP effect in young adults with high WMC was significant (*t*(8) = 3.54, p = .008), while the numerically negative difference in the low-WMC subgroup was not (*t*(10) = -1.82, p = 099; see Figs 2 and 3).

#### Early frontal old/new effects

We examined early frontal old/new effects between 300–500 ms as these appeared to differ between targets and non-targets, at least in the young group. The scalp distribution of the early frontal effects also appeared to differ between age groups (see Fig 1), with a central maximum in the young as expected, and a right sided maximum in the older. We therefore quantified them at 3 frontocentral electrode groupings rather than at the typical midline frontocentral sites, taking the mean across electrodes in each grouping (left: [FC3, FC5, FT7], midline: [FC1, FC2, FCz], right: [FC4, FC6, FT8]). ANOVA on old/new effects with factors of Group (young/older), Hemisphere (left/midline/right) and Condition (target/ non-target) revealed significant interactions of Group with Condition and with Hemisphere (*F*(1,38) = 11.65, MSE = 11.4, p = .002, η^2^_p_ = .24; *F*(1.9,73.4) = 3.94, MSE = 1.3, p = .025, η^2^_p_ = .09) as well as a significant intercept (indicating an old/new effect present across conditions and sites), as well as a main effect of Condition (*F*(1,38) = 7.01, MSE = 29.6, p = .012, η^2^_p_ = .16; *F*(1,38) = 8.99, MSE = 8.8, p = .005, η^2^_p_ = .19). *Post hoc* tests in the young showed a positive-going old/new effect only for non-targets, reflected in a main effect of Condition (*F*(1,19) = 19.92, MSE = 20.0, p < .001, η^2^_p_ = .51; for targets M = -.09 μV, *F* < 1; non-targets, M = .73 μV, *F*(1,19) = 7.15, MSE = 31.8, p = .015, η^2^_p_ = .27). In the older group the old/new effect was significant and positive-going but did not differ by condition (for targets M = .41 μV; non-target M = .38 μV; for intercept old/new effect, *F*(1,19) = 8.24, MSE = 17.6, p = .010, η^2^_p_ = .30; for Condition, *F* < 1).

ANOVA on rescaled data (see LP effect analysis for details of factors) confirmed that the topography of this effect differed according to age Group (for Group x Hemisphere, *F*(2.0,77.8) = 4.90, MSE = 12.4, p = .010, η^2^_p_ = .11, for Group x Chain, *F*(1.8,69.2) = 4.62, MSE = 12.7, p = .016, η^2^_p_ = .11; for 3-way interaction, *F*(1.4, 214.2) = 2.10, MSE = 1.4, p = .06). Effects involving Condition and Hemisphere or Chain were not significant. A *post hoc* test at the frontocentral electrode groupings confirmed a significant interaction of Group with Hemisphere (*F*(1.9,75.2) = 3.32, MSE = 3.4, p = .043, η^2^_p_ = .08). Because of this different topography between groups, we used data from the scalp maximum in each age group for the analysis of WMC effects (midline frontocentral electrodes in the young, right frontocentral electrodes in the older group). However, although the multiple regression model (with the same predictors as for the LP effect) was significant overall (*F*(4,30) = 4.6, MSE = 3.4, p = .005), this was driven by an intercept, i.e., a significant mean old/new effect; none of the predictors of interest were significant. Because of Sprondel et al.’s [33] prior finding of a positive relation between a frontal non-target old/new effect magnitude and O-span in young adults (but not adolescents), we also checked for a similar effect, but found no hint of any significant association in the present young group (*F*(1,18) = .03,MSE = 2.5, p = .87).

#### Late posterior negative old/new effects

In order to check whether age and WMC-related modulations of the LP effect were driven by the overlapping LPN (see Fig 1), we also examined these effects for the LPN between 800–1100 ms, i.e., outside the typical LP effect time window. The LPN was quantified at 3 centroparietal electrode groupings, taking the mean across electrodes in each grouping (left: (CP3, CP5, TP7), midline: (CP1, CPz, CP2), right: (CP2, CP4, TP8)). ANOVA on the old/new effect had factors of Group (young/ older), Hemisphere (left/ midline/ right), and Condition (target/ non-target) (excluding one older outlier). The results showed a midline maximum (for Hemisphere main effect, *F*(1.9,70.9) = 22.2, MSE = 42.57, p < .001, η^2^_p_ = .37; for intercept reflecting the average old/new effect, *F*(1,39) = 5.78, MSE = 76.9, p = .021, η^2^_p_ = .13). Interactions of Hemisphere with Condition and Hemisphere with Condition and Group were non-significant (*F*(2.0,74.3) = 3.08, *MSE* = 1.4, p = .053; *F*(2.0, 74.3 = 2.56, MSE = 1.7, p = .085) as were other Group effects (*F* < = 1.58).

As for the LP effect, we checked for differences in the scalp distribution of the LPN effect by age and condition prior to performing the regression analysis. ANOVA of rescaled data (see LP effect analysis for details of factors) showed a significant interaction of Hemisphere with Condition (*F*(1.9,74.3) = 5.14, *MSE* = 1.7, p = .009, η^2^_p_ = .12) reflecting in part a difference between conditions in the lateralization of the LPN (see also 3.3.1.4): at centroparietal sites, it had a midline maximum but a more right-sided distribution for non-targets (for Hemisphere x Condition, *F*(1.8,71.2) = 4.76, MSE = .75, p = .004, η^2^_p_ = .14). Scalp topography did not differ reliably according to age group (*F* < = 1.73).

Because of the difference in scalp topography between the LPN for targets and non-targets, the difference between its magnitude in these two conditions is difficult to interpret. Despite this, regression models were constructed to examine effects of age and WMC, in order to determine whether the age and WMC effects found for the LP effect were driven by the overlapping LPN. The dependent ERP old/new measures were taken from the midline parietal grouping where the LPN was maximal, and the dependent variable was again the difference in old/new effects for Targets—Non-targets. The model with predictors of Group, O-Span and the interaction of Group x O-Span, as well as Age, explained significant variance (R^2^ = .31, *F*(4,29) = 3.24, MSE = 16.7, p = .026) with an effect of O-Span (β = .45, *t*(33) = 2.64, p = .013) across both age groups, but no interaction of O-Span with Group (*t* < 1). *Post hoc* analysis of target and non-target effects separately showed no significant effects for the two trial types taken separately. However, the direction of the effect on the difference measure indicates that the pronounced negative target LPN tended to be smaller in participants with higher WMC.

#### Late frontal old/new effects

Following previous studies, we examined the late frontal old/new effect between 800–1100 ms. Inspection of Fig 1 suggested presence of a small effect, more visible in the older group. We quantified it across both groups at the 3 frontal electrode groupings, taking the mean across electrodes in each grouping (left: [F3, F5, F7, AF7], midline: [F1, F2, Fz], right: [F4, F6, F8, AF8]). ANOVA with factors of Group (young/ older), Hemisphere (left/ midline/ right), and Condition (target/ non-target) showed a significant interaction of Hemisphere with Condition (*F*(1.7, 65.4) = 4.50, MSE = 1.9, p = .019, η^2^_p_ = .11), reflecting a positive old/new effect over the right frontal scalp for targets only (for main effect of Hemisphere for targets, *F*(1.7, 67.1) = 9.29, MSE = 10.2, p = .001, η^2^_p_ = .19; for non-targets, *F*(1.7, 64.7) = 1.39, MSE = 1.4, p = .256). Group differences in the magnitude of this effect were not significant, and nor was the intercept (overall old/new effect). The intercept was also not significant in either group analyzed separately. Analysis of rescaled data in this time window had already revealed an interaction between Hemisphere and Condition across the scalp (see analysis of LPN, above). A separate analysis at frontal sites for targets only (since non-target frontal effects were non-significant) had the factor of Hemisphere. This confirmed a right frontal maximum in both groups (for Hemisphere, *F*(1.8, 70.5) = 9.88, MSE = 2.2, p < .001, η^2^_p_ = .20). The location of this maximum also differed between age groups, being more right-lateralized in the older group than in the young (see Fig S1, Supporting Information; for Group x Hemisphere, *F*(1.8, 70.5) = 3.42, MSE = .75, p = .043, η^2^_p_ = .08). However regression analysis for effects of individual differences in O-Span on the target-selectivity of the right frontal effects (see analysis of LP effects for details of model) was not significant (*F*(4,30) = 2.06, MSE = 4.3, p = .111).

## Discussion

The primary goal of this study was to examine the proactive control of episodic memory retrieval in aging. We evaluated the relations between individual differences in cognitive control beyond the memory domain and their hypothesized downstream effects on the prioritization of recollection, indexed in terms of the ERP left parietal effect for targeted compared to non-target remembered information. As predicted, recollection was selective for targeted information in young high-WMC adults and the degree of selectivity tracked individual differences in WMC, replicating earlier studies. WMC also correlated negatively with individual age in the older group. Although some older adults had high WMC and the groups did not differ on average in this regard, recollection in the older group showed no evidence of selectivity. Unlike in the young group there was no association between WMC and the degree of selectivity. Together, the data support the view that older adults are less able to prioritize recollection according to retrieval goals, and provide initial evidence linking a decline in this ability to their wider difficulties in cognitive control.

### Behavioral findings

The young and older groups showed typical performance on the standardized cognitive tests. Although the young adults had had more years of formal education, verbal IQ was greater in the older group, but accompanied by age-related reductions in processing speed and WMC. Processing speed differed between groups as expected, and it is important to note that although average WMC did not differ between groups in the present sample, it was correlated with age within the older group. Therefore, age-related effects on WMC were clearly present. The absence of the expected mean group difference in WMC is probably due to a combination of a wide range of both age and ability within the older group, with some relatively high-ability individuals, and a somewhat lower-ability young group than is typical in our work. In previous studies using closely similar tests of crystallized ability, mean verbal IQ has been closer between groups, although still lower in the young groups, reflecting lifespan accrual of knowledge (for example ([58]: young M = 114, older M = 119, ([59,60]: both young M = 112, older M = 118). However, we note that this limitation regarding the comparability of the groups is conservative with regard to our hypotheses, being likely if anything to lead to underestimation of group differences in episodic memory and in the relations between episodic memory and WMC. Moreover, while the fact that age effects on WMC were observed mainly within the older group complicated the analysis of age-related and WMC effects on ERPs (see ERP results), we were able to address this in our analysis, as discussed in detail below.

Exclusion task performance was high overall, well above chance for both sources, with mean target-non-target discrimination at over .6 in both tasks and both age groups, and somewhat greater accuracy on the Artist than the Function task across groups. The absence of age-related differences in overall level of performance indicates that our manipulation of task difficulty to match performance according to age was broadly successful. Therefore, age effects on ERPs cannot simply reflect the presence of overall poorer performance, for example contamination of successful trial activity with differential rates of ‘lucky guesses’ [61]]. Participants in both age groups were also better at rejecting novel items than identifying targets or rejecting non-targets, consistent with prior data (e.g., [62]]). However, there were subtle group differences in the pattern of performance for targets and non-targets, mainly in the Artist task. In that task, while the young were equally likely to correctly identify targets and reject non-targets, but slower to reject the non-targets, the older group were less likely, as well as slower, to correctly reject non-targets than to correctly identify targets. Thus for both age groups it was somewhat easier to accept Artist-studied targets than reject Function-studied non-targets, but this effect was more pronounced in the older group. In the Function task, group differences were minimal, present only for RTs. Both groups were faster to reject Artist-studied non-targets than identify Function-studied targets, but the older group also responded faster to non-targets than targets. Thus the older adults performed more poorly in the more difficult condition under each retrieval goal.

Importantly, the equivalent accuracy for the pre-studied target items suggest that the two groups did not differentially rely on recall-to-reject strategies. For these items, recall that they had been studied in the non-targeted task (as well as the targeted task) would lead to their (incorrect) rejection as non-targets (see Recognition memory task: Procedure).

We also assessed the relations between WMC and exclusion task performance in the two age groups. The results of this analysis were somewhat inconclusive, as the group difference in the relation between WMC and target-non-target discrimination by age group was non-significant. There was a strong association of WMC with better exclusion performance in the young group alone, and no hint of an association in the older group, consistent with the possibility that working memory capacity (or associated abilities; see below) was engaged to a greater degree or with greater effect by the young high-WMC participants in the episodic memory task, consistent with the ERP findings. Due to the non-significant group difference, we emphasize that this requires confirmation by future studies. Nonetheless, the data do confirm that among young adults, those with higher WMC were better able to distinguish targets from non-targets, as expected.

### ERP findings

#### Left parietal old/new effect

We examined the prioritization of recollection of targeted information, as reflected in the difference between target and non-target left parietal old/new effects. As predicted, the magnitude of this difference depended on age group and WMC. In higher WMC young adults, this difference was significant, and larger than in those with lower WMC, supporting the notion that there was relatively greater recollection of information about the currently targeted task. This differed from the older group, in whom there was no significant association between WMC and recollection selectivity, and no evidence of prioritization of recollection in the group as a whole (on average, the LP effect was numerically but not significantly larger for non-targets than targets; see lower panel of Fig 3). Findings in the young group generally replicate the earlier results of Elward et al. [28,30]. As in both the latter studies, the relation between WMC in healthy young adults also appeared to be driven mainly by an enhancement of LP effects for targets, suggesting that at least part of the ‘downstream’ effects of goal-directed cognitive control on recollection may be to emphasize recollection of targeted information, rather than to prevent or constrain recollection of non-targeted information.

The present age-related effects are in line with those reported by Dywan et al. [13] Experiment 1). In that study, older adults showed equivalent target and non-target LP effects while the young showed larger effects for targets. Our older adults are comparable to the previous study, but the young group as a whole did not show significant prioritization of recollection of targeted information, and the groups did not differ on average. This is likely because the younger group included some relatively low-performing individuals who did not show target selectivity in the LP effect (see Discussion of behavioral findings, above). Critically, although the age groups did not differ in this selectivity on average, the young adults showed a robust association between selectivity of the LP effect and individual differences in WMC as predicted. Furthermore, we found an age effect on the association between WMC and target-selectivity of LP effects, which was absent in the older adults, unlike the young. The results support our hypothesis that an age-related decline in the selectivity of recollection reflects impairment of wider cognitive control abilities, and we discuss the relation between WMC and these abilities in more detail below.

As noted in the Results, we included age as a continuous predictor in the regression models used to test for group differences in the relation between WMC and the target selectivity of ERP effects. This is important where age-ranges within groups are wide, in order to avoid confounding individual differences which change with age with average effects of age (see [56]] and ERP results section). It was particularly important in the present study where WMC varied significantly with age within the older group. A potential concern with including age in the model was that effects of WMC within the older group might also be discounted due to this correlation. However, a check analysis showed no evidence of this, since the effect of the age regressor was not significant, and the interaction of group with WMC was still highly significant without age in the model (see ERP results, Left parietal old/new effect).

One possible explanation for nonselective recollection in groups with reduced WMC is that they have generally poorer recollection than those with high WMC. Performance confounds are generally a concern in ERP studies where difficulty varies between groups, particularly in forced-choice tasks such as recognition memory where dilution of ERP effects by ‘lucky guesses’ will be greater in participants with poorer memory performance [61]]. This was one reason we chose to maximize comparability of the LP effects between groups by matching target recollection between the two groups. A second motivation for this manipulation was previous findings that increasing the probability of target recollection using a task manipulation can increase the degree to which target LP effects are larger than non-target LP effects [34,35]]. The matching of overall level of target hits across age groups means that age-related effects are unlikely to have been driven by different strategies adopted because of differences in overall difficulty. This view is bolstered by the fact that older and younger groups did not differ on average in the magnitude of target LP effects. Furthermore, as in Elward and Wilding’s [28]] study, there was no hint of a relationship between individuals’ probability of target hits and the ERP measure of the prioritization of recollection. This suggests that variable selectivity was unlikely to directly reflect the probability of accurate target identification, instead reflecting the degree to which cognitive resources could be mobilized to prioritize recollection of targeted over non-targeted information (see [28]]).

#### Early frontal old/new effects

In the present study we also observed early positive-going ERP old/new effects which were maximal over the frontocentral scalp, and differed between age groups and between targets and non-targets. These effects were present in the 300–500 ms time window typical of the ‘mid frontal’ old/new effects typically associated with familiarity (for review see [11]]; but also see [63,64] for discussion). As the present effects had a mid-frontal distribution only in the young group, and were right-lateralized in the older adults, it is difficult to interpret them straightforwardly in terms of familiarity, particularly in the older group. However, it is significant that the magnitude of the effect differed between targets and non-targets in the young group only, indicating divergence between the processing of targets and non-targets as early as 300 ms post-stimulus. The early old/new effect was present only for non-targets in the young, but for both conditions in the older group. In their developmental study, Sprondel et al. [33] observed an unexpected frontal positive-going old/new effect in their young adults which shared some features with the present findings, being present only for non-targets, although it occurred after the mid-frontal effect, overlapping temporally with their LP effect (500–700 ms). Finding that this effect was larger in higher-WMC young adults, the authors proposed that it reflected relatively early cue-specification processes used to discriminate between targets and non-targets. One possibility is therefore that the present early frontal effects indicate similar processes which discriminate between targets and non-targets only in young adults, although we did not find any association with WMC.

Alternatively, other early processes may enable young adults to detect non-targets and potentially to suppress their recollection. One way in which this could have occurred in the current study would be if the young adults modulated encoding processing based on the predictable order of the study-test cycles. However, we found no specific evidence for use of such a strategy, as performance did not improve over cycles (nor improve preferentially in the young adults), and a similar strategy could not have been adopted in Sprondel et al.’s study due to an irregular task sequence [33]. In Elward et al.’s [30] study similar results were obtained in young adults using only two study-test cycles, so prediction of retrieval goals could only have occurred on half the test trials. Furthermore, the early old/new effect findings are not consistent with the notion that WMC impacted selectivity of recollection via prioritization of *target* encoding, since such a strategy would be expected to emphasize early processing of targets rather than non-targets, the opposite to our pattern of findings. These possibilities cannot be distinguished on the basis of the present data, but the presence of an early ERP difference between target and non-target processing only in the young group is consistent with the general proposal that aging affects early goal-directed processing at retrieval, prior to the point of recollection.

#### Late old/new effects

To separate the LP effect from the late posterior negativity we measured it from 500–600 ms post-stimulus, omitting the latter part of the typical 500–800 ms time window. Older adults sometimes show later onset of ERP effects than the young, consistent with age-related slowing (e.g. [65], but in the present study a robust LP effect was present in both age groups in the selected time window. It was also left-lateralized at the *a priori* specified parietal electrodes (P5 and P6), consistent with typical findings in the young [11,12]. In older adults, parietal old/new effects are sometimes more bilateral, particularly when performance is relatively poor (e.g., [66–68]; see [69]). In the present study, scalp distribution of the old/new effect from 500–600 ms was age-invariant and did not differ between targets and non-targets.

The presence of a negative-going old/new effect from around 600–1100 ms post-stimulus is consistent with a class of effects referred to as the late posterior negativity, thought to reflect heterogeneous processes associated with recognition and source memory reconstruction [69–73]. The LPN most typically onsets after the LP effect but before the participant responds. Although this onset is generally rather later than the effect we observe here, we note that several prior studies have reported onsets around 600 ms (e.g., [71,74,75]), including our own [76]. However, in their recent review, Mecklinger and others [73] suggested that negative-going old/new effects in aging studies may not be true LPN effects, arguing from the tendency of these effects to be more centrally rather than posteriorly distributed over the scalp. It is therefore difficult to be sure of the functional significance of the effect in the present study, although we refer to it as an LPN for convenience.

The presence of this negative-going old/new effect in the present study raised the potential concern that the age- and ability-related associations with the LP effect were in fact driven by the overlap with this later effect, complicating the above interpretation of the findings. If overlap of the LP effect with the LPN were responsible for the results for the analyses at P5 between 500–600 ms, we expected a similar pattern of findings for the LPN alone. However, this analysis showed no hint of the group difference observed for the LP effect. Although presence of the overlapping LP effect did not drive the relation we observed between age, WMC and target-selectivity of the LP effect, it nonetheless a limitation of the present study that it was not possible to determine whether this relation was present for the entire duration of the LP effect. It may also be of interest that the magnitude of the LPN correlated with O-Span across both age groups. This suggests that whatever the cognitive operations indexed by this ERP difference are, these may be engaged in the further processing and evaluation of the products of recollection prior to the memory decision. Moreover, the direction of the effect suggested that while the LPN was larger for targets than non-targets overall, this negativity associated with identification of targets relative to new CR was less pronounced in participants with higher WMC. A possible explanation for this is that if recollection is more selective (reflected in the LP effect) then less additional processing (reflected in the LPN) is necessary. Our data suggest that such an effect may be age-invariant, indicating intact engagement of a more reactive element of retrieval control in older adults, consistent with our prior finding that older adults showed enhancement of an LPN-like effect in a source memory task [76]. However, the null group difference does not warrant strong interpretation. We also emphasize that because the scalp distributions of the negative-going old/new effects differed between conditions, interpretation of their difference measure is uncertain. Finally, in the same time window, we found no evidence that the processes reflected in the positive-going late right frontal old/new effect were differentially engaged by young adults or those with higher WMC.

### Aging, recollection selectivity and cognitive control

The current data, together with the previous findings of Elward and others [28,30], provide insights into how working memory capacity supports the prioritization of recollection of goal-relevant over goal-irrelevant content, and how this selection is impaired in aging. The findings in older adults are closely similar to those reported by Sprondel et al. [33] in adolescents, and by Elward et al. [30] in healthy young adults who had previously performed a taxing Stroop interference task. The impact of this Stroop intervention specifically supported the proposal that cognitive control functions indexed by measures of WMC are critical for the prioritization of recollection according to current goals. However, our data are not consistent with the simple view that reduction of WMC in older adults directly impairs their ability to select what will be recovered from memory. Instead, they suggest that with aging there is an uncoupling between WMC and recollection selectivity. Even though many of the present older group performed as well as the young on the O-Span task, it appears that—unlike in the young—the abilities reflected in these scores did not support effective pre-retrieval control in this recognition task requiring controlled recollection. This suggests that maintaining working memory capacity is insufficient to enable older adults to effectively prioritize recollection in line with current goals. The similarity between this result and those of Sprondel et al. [33] in adolescents further suggests that other populations with reduced cognitive control ability may show a similar uncoupling.

The precise locus of the uncoupling between WMC and prioritization of recollection in aging is currently unknown, and cannot be established on the basis of the present data. Our understanding of the interrelations between WMC, recollection, and cognitive control beyond the memory domain in young adults is still evolving (see [22–24,77]. However, the current results suggest that the contribution of WMC to the prioritization of targeted information in episodic retrieval is mediated by additional processes which are impaired in aging. As outlined in the Introduction, strong candidates for these additional processes are those involved in proactive attentional control. Working memory capacity indexes the ability to maintain goal information online in the presence of interference, and in young adults, higher WMC is associated with greater use of proactive control on the AX-CPT task [23,24]. As noted in the Introduction, older adults show impairments in proactive control on tasks such as the AX-CPT, and greater reliance on reactive control [17–21]. Importance of proactive control in recollection is suggested by studies of free-recall which show that higher-WMC young adults are better at targeting the recollection of specific information, and more able to self-generate retrieval cues and engagement of appropriate retrieval strategies, as well to maintain the cues in working memory (see [77] for review). Sprondel et al. [33] suggested that WMC was not related to the prioritization of recollection in their adolescent group because development of cue-specification processes was incomplete. Similarly, the effect of aging on the prioritization of recollection may stem mainly from impairment of proactive control itself, impacting the pre-retrieval control processes such as the self-generation of retrieval cues and strategies, rather than directly reflecting age-related reduction in WMC. If this proposal is correct, independent influences of age should be detectable on WMC and measures of proactive cognitive control, and in older adults proactive control will correlate with the prioritization of recollection, although WMC does not. Further studies are also needed to understand how and when pre-retrieval control processes such as cue specification are impaired in older adults, and to establish their timing in relation to recollection (see [9], as well as to identify in young adults the precise ways in which both WMC and proactive attentional control support the prioritization of recollection.

## Conclusions

The data presented here support the view that aging impairs the ability to select what is recovered from episodic memory [9]. Together, the results support the notion that although WMC is important for the prioritization of recollection, reduced WMC in older adults is not the direct determinant of this impairment. They extend the earlier ERP findings of Dywan et al. [13] which suggested that recollection is less selective in older than young adults, and provide a first link with wider age-related difficulties in proactive cognitive control.
